# Assembly and Budding of *Ebolavirus*

**DOI:** 10.1371/journal.ppat.0020099

**Published:** 2006-09-29

**Authors:** Takeshi Noda, Hideki Ebihara, Yukiko Muramoto, Ken Fujii, Ayato Takada, Hiroshi Sagara, Jin Hyun Kim, Hiroshi Kida, Heinz Feldmann, Yoshihiro Kawaoka

**Affiliations:** 1 Laboratory of Microbiology, Department of Disease Control, Graduate School of Veterinary Medicine, Hokkaido University, Sapporo, Japan; 2 International Research Center for Infectious Diseases, Institute of Medical Science, University of Tokyo, Shirokanedai, Minato-ku, Tokyo, Japan; 3 Core Research for Evolutionary Science and Technology, Japan Science and Technology Corporation Agency, Saitama, Japan; 4 Division of Virology, Department of Microbiology and Immunology, University of Tokyo, Shirokanedai, Minato-ku, Tokyo, Japan; 5 Fine Morphology Laboratory, Department of Basic Medical Science, Institute of Medical Science, University of Tokyo, Shirokanedai, Minato-ku, Tokyo, Japan; 6 Department of Pathobiological Sciences, School of Veterinary Medicine, University of Wisconsin-Madison, Madison, Wisconsin, United States of America; 7 Special Pathogens Program, National Microbiology Laboratory, Public Health Agency of Canada, Winnipeg, Manitoba, Canada; 8 Department of Medical Microbiology, University of Manitoba, Winnipeg, Manitoba, Canada; Washington University School of Medicine, United States of America

## Abstract

*Ebolavirus* is responsible for highly lethal hemorrhagic fever. Like all viruses, it must reproduce its various components and assemble them in cells in order to reproduce infectious virions and perpetuate itself. To generate infectious *Ebolavirus,* a viral genome-protein complex called the nucleocapsid (NC) must be produced and transported to the cell surface, incorporated into virions, and then released from cells. To further our understanding of the *Ebolavirus* life cycle, we expressed the various viral proteins in mammalian cells and examined them ultrastructurally and biochemically. Expression of nucleoprotein alone led to the formation of helical tubes, which likely serve as a core for the NC. The matrix protein VP40 was found to be critical for transport of NCs to the cell surface and for the incorporation of NCs into virions, where interaction between nucleoprotein and the matrix protein VP40 is likely essential for these processes. Examination of virus-infected cells revealed that virions containing NCs mainly emerge horizontally from the cell surface, whereas empty virions mainly bud vertically, suggesting that horizontal budding is the major mode of *Ebolavirus* budding. These data form a foundation for the identification and development of potential antiviral agents to combat the devastating disease caused by this virus.

## Synopsis

*Ebolavirus* is a causative agent of a severe, mostly fatal hemorrhagic fever in humans. Like all viruses, it must reproduce its various components and assemble them to reproduce progeny virus particles. However, because *Ebolavirus* needs special containment for biosafety, progress in understanding its morphogenesis has been slow, resulting in no effective therapies currently available for humans. To further our understanding of the *Ebolavirus* life cycle, the authors examined the specific interactions among viral proteins and the processes of *Ebolavirus* morphogenesis.

It is demonstrated that nucleoprotein likely serves as a core for the nucleocapsid (NC) complex, which is critical for replication and transcription of viral genome. The interaction of nucleoprotein with matrix protein VP40 is found to be essential for transport of NCs to the cell surface and for the incorporation of NCs into virions, resulting in the formation of mature virus particles. Unique among all viruses to our knowledge, *Ebolavirus* particles containing NCs mainly emerge horizontally from the cell surface, whereas the other viruses bud vertically. These findings form a foundation for the identification and development of potential antiviral agents to combat the devastating disease caused by this virus.

## Introduction

*Ebolavirus,* together with *Marburgvirus,* comprise the family Filoviridae in the order Mononegavirales [1,2]. It has a nonsegmented, negative-sense RNA genome that encodes at least seven structural proteins [1,3]. These proteins form filamentous particles 80 nm in diameter with lipid membrane derived from host cells. The viral glycoprotein (GP) protrudes from the surface of the viral envelope, while the matrix protein VP40 plays a central role in the morphogenesis of the filamentous virions [4–7]. Along the central axis of the filamentous virion resides a nucleocapsid (NC) of approximately 50 nm in diameter. This viral genomic RNA-protein complex has an axial channel at its center [8]. The NCs of *Ebolavirus,* which represent the principal units of transcription and replication of the viral genome, are thought to consist of four proteins: the L polymerase protein, VP35, nucleoprotein (NP), and VP30. Huang et al. [9] showed that expression of NP, the membrane-associated VP24 protein, and VP35 results in the formation of structures morphologically indistinguishable from the NCs observed in *Ebolavirus*-infected cells, demonstrating the involvement of VP24 in the formation of such structures.

In *Ebolavirus* infection, newly synthesized viral proteins and genomic RNA in the form of NCs are transported to the budding site where the viral components assemble to form virions [7,10–13]. However, many questions regarding assembly of *Ebolavirus* particles have yet to be answered. How are NCs formed in the cytoplasm? How are NCs transported to the cell surface and incorporated into virions? How do the filamentous virions bud from the cell surface? In an attempt to answer these questions, we performed structural and biochemical assessments of cells transfected with plasmids expressing various combinations of Ebola viral proteins and of cells infected with *Ebolavirus.*

## Results/Discussion

### Formation of NC-Like Structures

**Figure 1:**
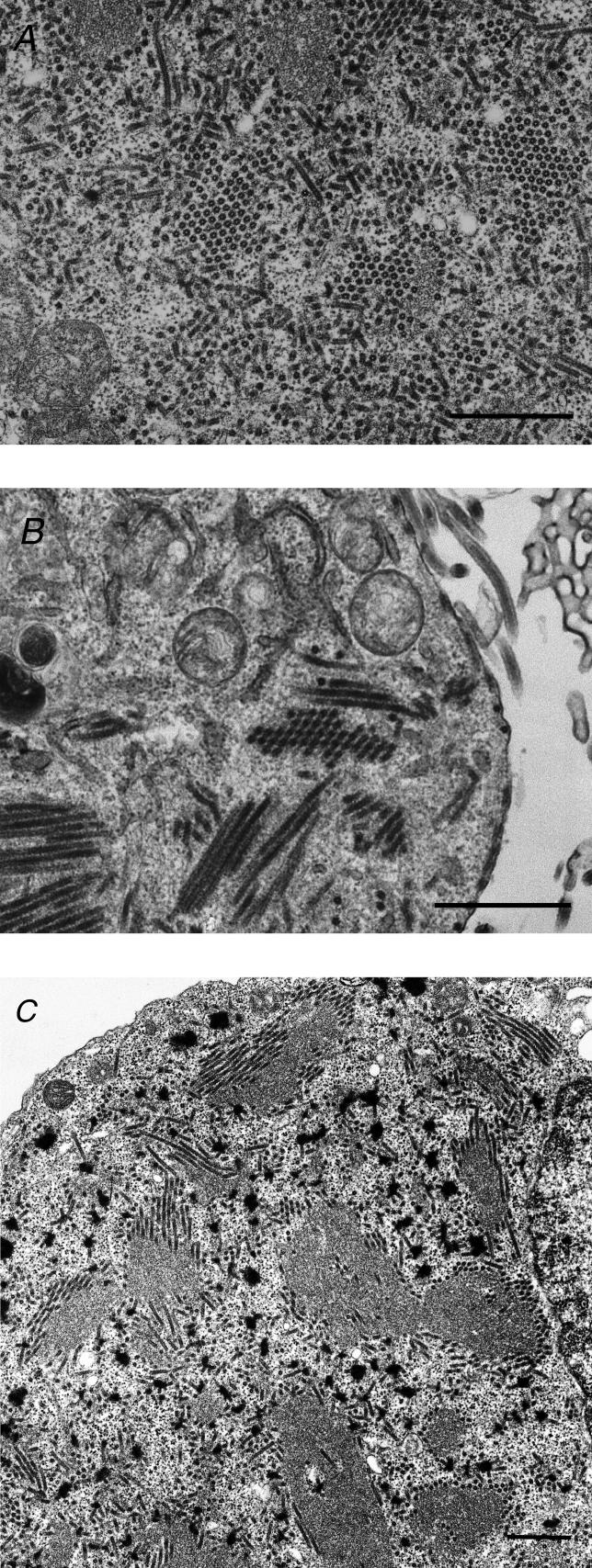
Formation of NC-Like Structures upon the Expression of NP, VP24, and VP35 (A) Expression of NP, VP24, and VP35 produced filamentous and tubular NC-like structures approximately 50 nm in diameter. (B) In *Ebolavirus*-infected cells, a large number of NCs newly synthesized in the cytoplasm were observed. (C) When NP, VP24, and VP35 were expressed simultaneously, NC-like structures were found at the edge of the NP tubes. Bars, 1 μm (A, B, and C).

To confirm whether the expression of NP, VP24, and VP35 are sufficient for the formation of NC-like structures [9,14], we cotransfected 293T cells with various combinations of plasmids for the expression of the viral proteins (i.e., NP, VP24, VP30, VP35, and L) and the minigenome RNA, which consists of a green fluorescent protein gene flanked by 3′ leader and 5′ trailer sequences. As previously described [9,14], coexpression of NP, VP24, and VP35 was indispensable for the formation of NC-like structures (Figures 1A and S1A) that are morphologically indistinguishable from NCs in virus-infected cells (Figure 1B), whereas the viral minigenome RNA was not essential for this process. To understand the roles of the individual proteins in the formation of the NC-like structures, we transfected cells with plasmids expressing NP, VP24, or VP35. When NP was expressed alone, helical tubes, the diameter of which was almost the same as that of a central portion of the NCs (approximately 20–25 nm in diameter), possessing a central channel (approximately 15–20 nm in diameter), were arranged in a bundle in the cytoplasm (Figure S1B–S1D). In the cytoplasm of VP35-expressing cells, large (approximately 8-μm × 3-μm) electron-dense structures with small opaque areas (less than 200 nm in diameter) were observed near the nucleus (Figure S1E). By contrast, in cells transfected with the VP24 plasmid, numerous, small electron-dense pleiomorphic aggregates, identified as VP24 by immunoelectron microscopy with an anti-VP24 antibody (unpublished data), were seen scattered throughout the cytoplasm (Figure S1F).

To understand how NC-like structures are formed, we coexpressed NP, VP24, and VP35 in different combinations. On coexpression of VP35 with VP24, we saw a large number of pleiomorphic particles near the nucleus that were different from the VP35- or VP24-induced structures (Figure S1G), although an interaction between VP35 and VP24 was not detected by a coimmunoprecipitation assay (unpublished data). When VP35 was expressed with NP, small pleiomorphic structures, whose electron density was similar to that of the VP35-induced structures, were observed at the periphery of the clusters formed with NP tubes (Figure S1H). By contrast, when VP24 was expressed with NP, the morphologies of the small pleiomorphic structures formed by VP24 and of the NP tubes did not change (unpublished data). Finally, when the three proteins were expressed simultaneously, NC-like structures approximately 50 nm in diameter were found at the edge of the clusters of NP tubes (Figure 1C). These results, together with the previous biochemical studies demonstrating interactions between NP and VP24 *(Ebolavirus)* or NP and VP35 *(Ebolavirus* and *Marburgvirus)* [9,15], suggest that NP helical structures likely serve as the core for the formation of the NC-like structures, and that VP35 and VP24 contribute to this process by interacting with NP at the periphery of the NP clusters.

### VP40 Is Critical for the Transport of NC-Like Structures and for Virion Incorporation

**Figure 2:**
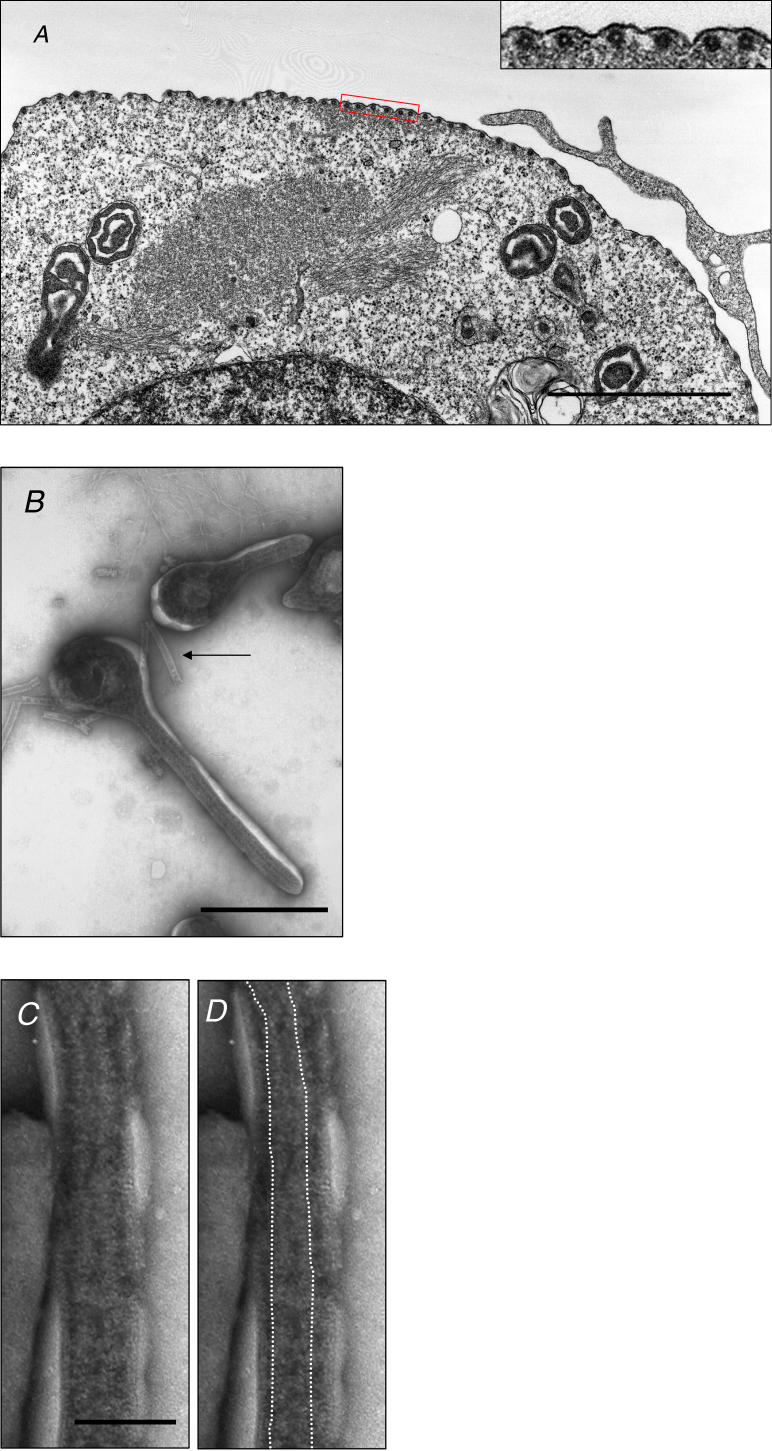
VP40 Expression Is Essential for the Transport of NC-Like Structures to the Plasma Membrane (A) A micrograph of the transport of NC-like structures in cells expressing NP, VP24, VP35, and VP40. (A, inset) Enlargement of the boxed area depicting the cross section of six NC-like structures beneath the plasma membrane. (B) NC-like structures were incorporated into VLPs. Inside the VLPs, tubular NC-like structures are observed. NC-like structures released from VLPs broken during sample preparation can also be seen in the same field (arrow). (C and D) An NC-like structure (indicated by broken lines in D) residing along the central axis of a filamentous VLP. Bars, 2μm (A), 500 nm (B), or 100 nm (C).

Upon expression of NP, VP35, and VP24, NC-like structures were not found at the plasma membrane where *Ebolavirus* buds off, indicating that the formation and transportation of NCs to the cell surface are separate events and likely independently regulated. To determine which viral proteins are required for NC transport to the cell surface, we expressed the viral proteins involved in the formation of NC-like structures, other viral proteins (i.e., L, VP30, VP40, and GP), and the minigenome RNA in 293T cells. When VP40 was coexpressed with the proteins required for the NC-like structures (i.e., NP, VP24, and VP35), these structures were found immediately beneath the plasma membrane (Figure 2A) in an orderly arrangement, even in the absence of other viral proteins (i.e., L, VP30, and GP) and the minigenome viral RNA (unpublished data). Furthermore, VP40 proteins were detected by immunoelectron microscopy near the NC-like structures underneath the plasma membrane (Figure S2A). However, without VP40, NC-like structures at the plasma membrane could not be detected (Figure S2B). These results indicate that VP40 plays an important role in the transport of NC-like structures to the cell surface.

Expression of VP40 results in the formation of virus-like particles (VLPs), which are released from plasmid-transfected cells [4,5]. To determine whether NC-like structures are incorporated into VLPs, we examined the VLPs released from cells expressing NP, VP24, VP35, and VP40 by negative-staining electron microscopy. Smooth-surfaced, filamentous VLPs were found in the supernatants (Figure 2B). In most of the VLPs, NC-like structures were present along the central axis (Figure 2C and 2D), as is seen in NCs detected in authentic Ebola virions [8]. These observations indicate that VP40 alone is sufficient for NC incorporation into virions and that the surface membrane GP is not required for this event, unlike influenza virus glycoproteins [16].

### VLP Budding Is Dependent on Microtubules

**Figure 3:**
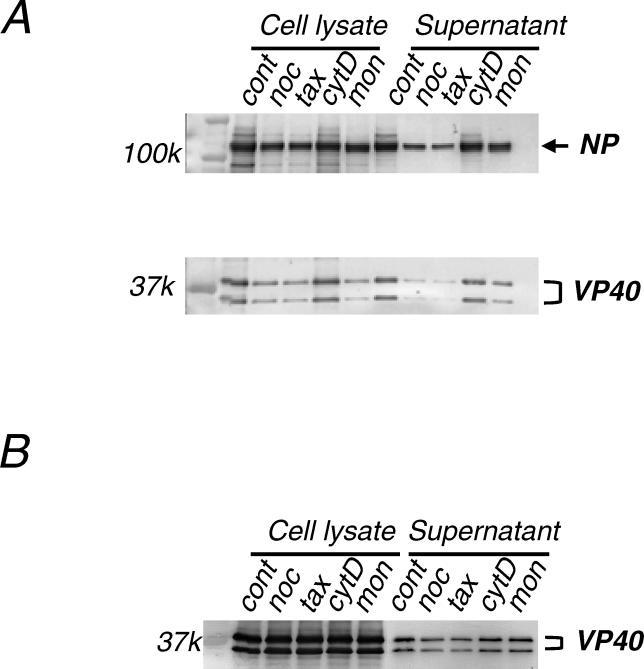
VLP Budding Is Dependent on Microtubules 10 μM nocodazole (noc), 1 μM taxol (tax), 10 μg/ml cytochalasin D (cytD), or 5 μM monensin (mon) was added to cells 3 h after they were transfected with plasmids expressing (A) NP, VP24, VP35, and VP40, or (B) VP40 alone. At 16 h post-transfection, proteins in the cell lysates and supernatants were separated by SDS-PAGE and examined by Western blotting with anti-NP and anti-VP40 antibodies. Following nocodazol or taxol treatment, the amounts of both VP40 and NP (A) or VP40 (B) in the supernatants (i.e., efficiency of VLPs budding) were reduced. cont, mock-treated control.

To determine which cellular components are involved in the transport of the NC-like structures to the plasma membrane, we expressed NP, VP24, VP35, and VP40 in cells treated with an intracellular vesicular trafficking inhibitor (monensin), an actin polymerization inhibitor (cytochalasin D), a microtubule polymerization inhibitor (nocodazole), or a microtubule depolymerization inhibitor (taxol). Even when actin polymerization was disturbed by 10 μg/ml cytochalasin D (the effect of the drug was confirmed by a change in enhanced yellow fluorescent protein [EYFP-β] actin distribution visualized by fluorescence microscopy; not shown), the amounts of VP40 and NP detected in the supernatants of the drug-treated cells did not differ from those of untreated cells (Figure 3A), despite a previous report suggesting the involvement of actin in virion formation [17]. When vesicular transport was inhibited by 5 μM monensin (a condition under which the transport of the vesicular stomatitis virus glycoprotein was confirmed to be inhibited [18]; unpublished data), a significant effect on release of VP40 and NP was not observed (Figure 3A). By contrast, perturbation of the microtubule structures by either 10 μM nocodazole or 1 μM taxol (as indicated by changes in EYFP-tubulin distribution; not shown) led to a reduction in the levels of NP and VP40 detected in the culture supernatants by more than 56% for NP and more than 57% for VP40 (Figure 3A).

Using electron microscopy we next examined whether microtubule-perturbating drugs inhibit the transport of NC-like structures to plasma membrane. In the presence of nocodazole or taxol, we did not detect an appreciable difference in the intracellular localization of NC-like structures, by comparison to localization in the absence of these drugs (unpublished data). We therefore tested the effect of these drugs on VLPs produced by VP40 alone. We found that perturbation of microtubules with these drugs reduced VP40-induced VLP release into culture media by 62% for nocodazole and 64% for taxol (Figure 3B). This finding is consistent with a recent report that *Ebolavirus* VP40 directly associates with microtubules [19]. These data suggest that a microtubule-dependent pathway may be involved in the release of VLPs from cells, which likely explains the concomitant reduction of NC-like structures in the culture supernatant.

### Interaction between NP and VP40 during the Formation of VP40-Induced VLPs

When VP40 and NP are coexpressed, NP migrates beneath the plasma membrane [20], and large amounts of NP are detectable in the supernatant [21]. To make certain that the NP detected in the supernatant was indeed incorporated into VLPs and not released as free NP, we performed a floatation analysis with the supernatants of cells expressing either NP alone or NP and VP40. When NP was coexpressed with VP40 (Figure S3A), it was detected in fractions of low sucrose concentration (i.e., approximately 1.12 g/cm^3^, fraction number 3) and as free protein in fractions of higher sucrose concentration. However, when NP was expressed alone, it was not detected in the supernatant of the transfected cells (Figure S3B). These results suggest that NP exists in membrane-bound form in the supernatant of cells that express both NP and VP40.

**Figure 4:**
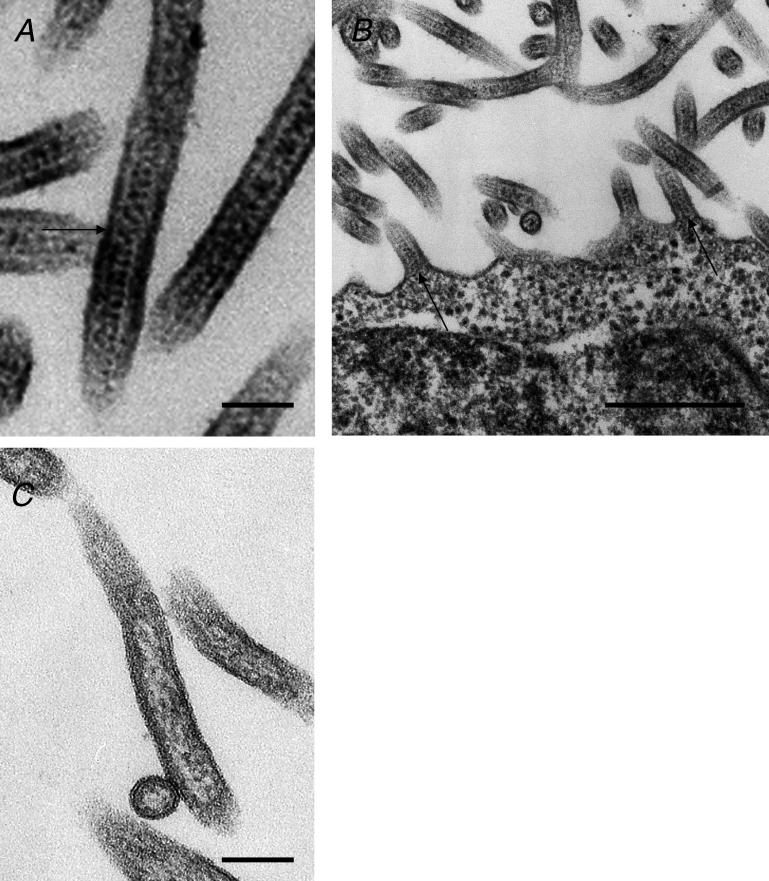
NP Helices Are Incorporated into VLPs (A and B) In cells coexpressing NP and VP40, NP helices can be seen in newly released VP40-induced VLPs (A, arrow), as well as in VLPs just about to bud (B, arrows). (C) Empty VLPs released from cells expressing VP40 alone. Bars, 100 nm (A and C) or 500 nm (B).

To obtain direct morphologic evidence that NP is incorporated into VLPs, we used transmission electron microscopy (TEM) to examine cells coexpressing NP and VP40. Following coexpression of NP and VP40, we observed filamentous VLPs containing NP helical structures (Figure 4A), which were not observed in VLPs produced by the expression of VP40 alone (Figure 4C). Indeed, helical NP structures were found even in VLPs that were just about to bud (Figure 4B, arrows). To demonstrate a direct interaction between VP40 and NP, we transfected 293T cells with a VP40-expressing plasmid alone or together with an NP-expressing plasmid and subjected the cell lysates and supernatants to immunoprecipitation with appropriate antibodies. VP40 coimmunoprecipitated with NP, and conversely, NP with VP40, demonstrating the direct interaction between these two proteins (Figure S4A and S4B). These results strongly suggest that the interaction between NP and VP40 is responsible for the incorporation of NCs into virions.

### *Ebolavirus* Buds in Two Distinct Modes

**Figure 5:**
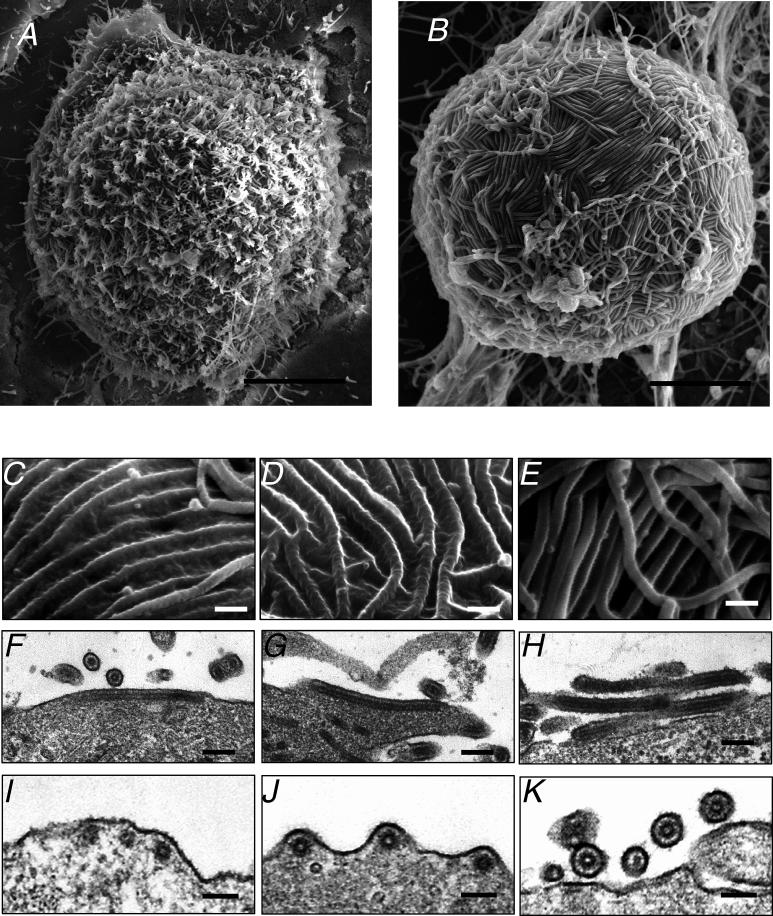
Different Modes of *Ebolavirus* Budding (A) Filamentous virions, approximately 80 nm in diameter, budding from the cell surface vertically or (B) horizontally. (C, F, and I, early stage; D, G and J, intermediate stage; E, H, and K, final stage). (C–E) The various steps of horizontal budding observed by SEM. Longitudinal (F–H) and transverse (I–K) sections of virions at different stages of horizontal budding visualized by TEM. Bars, 3 μm (A and B), 200 nm (C–H), or 100 nm (I–K).

To gain insight into the budding process of *Ebolavirus,* we examined Vero E6 cells infected with *Ebolavirus* by scanning electron microscopy (SEM) 2 d post-infection. Unlike uninfected Vero E6 cells (Figure S5A), most of the *Ebolavirus*-infected cells “rounded up” (Figure S5B), most likely because of GP-mediated downregulation of cellular adhesion molecules, including integrins [22,23]. In these virus-infected cells, which harbored many virions on their surface, two different modes of virus budding were observed. Filamentous virions were released vertically from the cell surface (Figure 5A), as previously documented for other filamentous virions [24–26], but, in addition, many virions emerged horizontally through the plasma membrane (Figure 5B). In our studies, both types of budding were rarely found in the same cells. Since horizontally budding viruses have never been reported previously, we further studied these horizontally budding virions by both SEM and TEM. At an early stage of horizontal budding, an orderly array of filaments began to emerge (Figure 5C, 5F, and 5I). As the horizontal budding progressed further (Figure 5D, 5G, and 5J), the membranes of the pre-virions were still connected to the cell. The filamentous Ebola virions eventually broke off, completing the budding process (Figure 5E, 5H, and 5K). These results show that filamentous Ebola virions do indeed bud from the plasma membrane horizontally.

### Mature Ebola Virions Containing NCs Primarily Bud Horizontally

**Figure 6:**
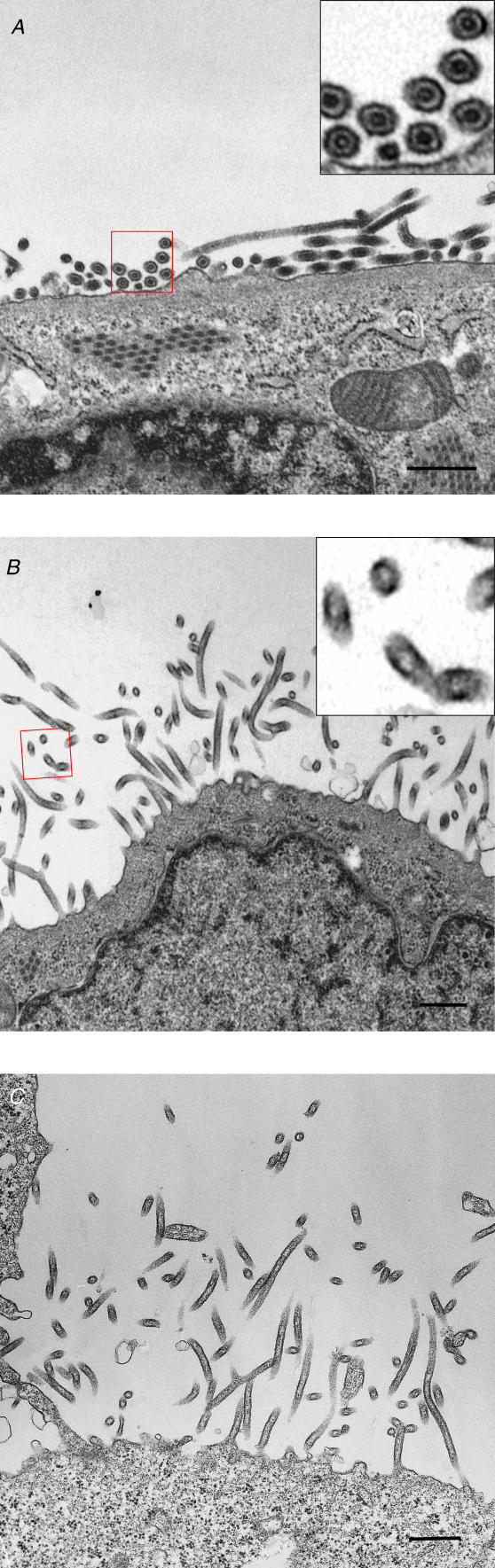
Virions Showing Two Different Modes of Budding (A) The majority of the virions is emerging horizontally from the cell surface and contains nucleocapsids (B), whereas vertically budding virions appear empty. Insets in (A) and (B) are larger magnifications of the areas identified in boxes of the main pictures, depicting cross sections of VLPs. (C) VLPs induced by the expression of VP40 alone bud vertically and lack NC-like structures. Bars, 400 nm.

To establish the prevalence of these two modes of *Ebolavirus* budding, we randomly selected virus-infected cells, 2 d post-infection, that had rounded up and were producing virions (*n* = 123) and used SEM to determine what percentage were covered with horizontally budding virions. More than 80% of the cells were producing virions horizontally, indicating that horizontal budding is the dominant mode of *Ebolavirus* budding. What determines the mode of *Ebolavirus* budding? One possibility is the presence of NCs and their interaction with the matrix protein VP40. Support for this hypothesis comes from our previous observation that expression of VP40 produces VLPs that lack NCs [5], and that these VLPs bud vertically (Figure 6C). For this reason, we examined the NCs inside particles budding horizontally or vertically. Most of the horizontally budding virions contained NCs. Empty particles were rarely found (Figure 6A). By contrast, although some of the vertically budding virions contained NCs, most were empty particles and had slightly smaller diameters than the horizontally budding virions that contained NCs, likely because of the lack of NCs (Figure 6B). We counted more than 100 virus particles containing NC (*n* = 109) in ultrathin sections to determine the ratio of horizontally budding virions to vertically budding virions. 98.2% of the virus particles possessing NC inside the particles emerged horizontally from the cell surface. Therefore, we conclude that Ebola virions possessing NCs mainly bud horizontally.

Since the matrix proteins of many negative-strand RNA viruses are known to interact with their NCs during assembly [27], it is conceivable that the NCs and the matrix proteins of *Ebolavirus* affect the budding mode of virions. Because *Ebolavirus* NCs synthesized in the cytoplasm are conveyed to the plasma membrane by a mechanism involving VP40 (Figure 2A), it may be that the empty VLPs induced by VP40 are released vertically because they did not accumulate enough NCs in the cytoplasm.

**Figure 7:**
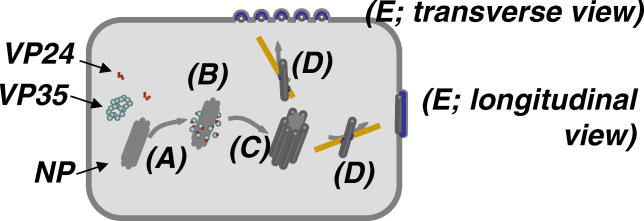
A Model of Ebola Virion Formation (A) NP self-assembles to form helical structures. (B) VP35 and VP24 interact with the helical structures formed by NP, resulting in the formation of NC-like structures (C). (D) NC-like structures are transported to the plasma membrane in the presence of VP40 via a microtubule-dependent pathway. (E) NC-like structures are incorporated into VLPs through an interaction between VP40 and NP that results in the production of filamentous particles budding horizontally from the cell surface.

Our findings, together with previous biochemical data [9,15], suggest a model for Ebola virion formation (see Figure 7): NP self-assembles to helical tubes. VP35 and VP24 possibly interact with NP around the mass of NP tubes, resulting in the formation of NC-like structures. The NC-like structures are then transported to the cell surface via a microtubules-dependent pathway mediated by VP40. Finally, the NCs are incorporated into virions through an interaction between NP and VP40 and bud mainly horizontally from the cell surface. Thus, our study provides morphologic evidence of interactions among viral proteins during the processes of virion assembly and budding. These results clearly further our understanding of the *Ebolavirus* life cycle. More detailed investigation should provide attractive targets for the development of antiviral compounds, such as inhibitors of virion assembly, formation, or budding.

## Materials and Methods

### Cells and virus.

293T cells and Vero E6 cells were maintained as described previously [28]. Vero E6 cells were infected with Zaire ebolavirus at a multiplicity of infection of 0.5. All infectious materials were handled in the biosafety level 4 facility of the National Microbiology Laboratory, Public Health Agency of Canada, at the Canadian Science Center for Human and Animal Health, Winnipeg, Manitoba, Canada.

### Plasmids and cell transfection.

All Zaire ebolavirus cDNAs were cloned separately into the mammalian expression vector pCAGGS/MCS [29,30]. A plasmid expressing an *Ebolavirus* minigenome, which contains a green fluorescent protein (GFP) gene flanked by 3′ leader and 5′ trailer sequences was described elsewhere [28,31]. 293T cells grown in 6-well plates were transfected with plasmids using Trans IT 293 reagent (Mirus Corporation, Madison, Wisconsin, United States).

### Chemical reagents.

Cytochalasin D, nocodazole, taxol, and monensin sodium salt were purchased from Sigma-Aldrich (St. Louis, Missouri, United States). Final concentrations of 10 μM Nocodazole (noc), 1 μM taxol (tax), 10 μg/ml cytochalasin D (cytD), or 5 μM monensin (mon) were added to culture media 3 h post-transfection of plasmids, and samples were harvested 16 h post-transfection.

### Western blotting.

293T cells transfected with protein expression plasmids were lysed in an SDS-PAGE (sodium dodecyl sulfate-polyacrylamide gel electrophoresis) sample buffer, and separated on a 4%–20% PAGE Tris-glycine gradient gel (Daiichi Pure Chemicals Company, Tokyo, Japan). Blots were then incubated with rabbit anti-NP and anti-VP40 serum, and binding of the primary antibodies was detected with the Vectastain ABC kit (Vector Laboratories, Incorporated, Burlingame, California, United States). Bands were then visualized by immunostaining (HRP-1000, Konica Minolta Holdings, Incorporated, Tokyo, Japan), and their relative intensities calculated by using Scion Image (http://www.scioncorp.com).

### TEM and SEM.

TEM and immuno-TEM were carried out as described previously [5]. More than 50 transfected cells were examined for each test sample. For negative staining, the culture supernatant of 293T cells was stained with 5% uranyl acetate. All samples described above were examined with a JEM-1200EX electron microscope at 80 kV. SEM was performed as described previously [28].

### Fluorescence microscopy.

To determine the effects of drugs (nocodazole, taxol, and cytochalasin D) on NC-like structure transport and incorporation into virions, 293T cells were transfected with 0.5 μg of pEYFP-actin expressing enhanced yellow fluorescence protein, (EYFP)-β actin fusion protein, or pEYFP-tublin expressing EYFP-α actin fusion protein (Becton Dickinson, Franklin Lakes, New Jersey, United States) and then treated with the drugs 3 h post-transfection. At 16 h post-transfection, the EYFP proteins were visualized with a BZ-8000 fluorescence microscope (Keyence, Osaka, Japan).

### Floatation assay.

Supernatants of plasmid-transfected 293T cells grown in 6-well plates were concentrated to 500 μl with Amicon Ultra-4 (Millipore, Billerica, Massachusetts, United States) and mixed with 1.5 ml of 80% sucrose. They were then overlaid with 2 ml of 50% sucrose and 1.2 ml of 10% sucrose in SW55 centrifuge tubes, subjected to centrifugation in a Beckman SW55 rotor at 250,000 × *g* at 4 °C for 16 h, and fractionated (0.6 ml/fraction) from the top.

### Immunoprecipitation assay.

293T cells were transfected with a plasmid expressing VP40 alone or together with an NP-expressing plasmid, and 48 h later lysed in TNE buffer (10 mM Tris-HCl [pH 7.8], 1% NP40, 0.15M NaCl, 1 mM EDTA). Supernatants of similarly transfected cells were also harvested 48 h post-transfection, concentrated by using Amicon Ultra-4 (Millipore), and then lysed in the same buffer. After clarification by centrifugation, the supernatants were incubated with a rabbit anti-NP antibody (1:500 dilution). Immune complexes were precipitated by incubation with protein G sepharose beads (New England Biolabs, Incorporated, Beverly, Massachusetts, United States), which were suspended in the sample buffer. After removal of the sepharose beads, the samples were subjected to SDS-PAGE, followed by Western blot analysis with a rabbit anti-VP40 antibody. For reciprocal immunoprecipitation assays, cells were transfected with a plasmid for expression of NP alone or together with a plasmid for expression of FLAG-tagged VP40, and the assays were performed with cell lysates and the VLPs in the culture supernatants.

## Supporting Information

Figure S1 Electron Micrographs of Cells Expressing NP, VP24, and VP35 (A) Coexpression of NP, VP24, and VP35 resulted in the formation of NC-like structures.
(B–D) Expression of NP alone produced a mass of helical tubes (C, dotted line) approximately 20–25 nm in diameter near the nucleus (Nuc) (B). Transverse sections of the NP helices are seen on the right (D), while longitudinal sections can be seen on the left (D).
(E) Expression of VP35 alone formed large electron-dense aggregates (arrow) near the nucleus (nuc).
(F) Expression of VP24 alone resulted in numerous small pleiomorphic structures (arrows) near the nucleus (nuc).
(G) Coexpression of VP35 and VP24 produced large structures near nucleus (nuc) that differed from the aggregates formed by expression of either VP24 or VP35 alone.
(H) Accumulation of electron-dense aggregates around the mass of NP tubes (arrows) was observed in the cytoplasm of cells coexpressing NP and VP35. Bars, 50 nm (A and B), 2 μm (C, E, G, and H), 500 nm (D), or 1 μm (F).
(713 KB PDF)

Figure S2 VP40 Is Essential for the Transport of NC-Like Structures to the Cell Surface (A) In cells expressing NP, VP24, VP35, and VP40, VP40 was detected by an anti-VP40 antibody conjugated with 15 nm gold, near to the NC-like structures (white arrows) located beneath the plasma membrane.
(B) Upon expression of all viral proteins except VP40 and the minigenome viral RNA, NC-like structures (arrows) remained in the cytoplasm. Bars, 100 nm (A) or 1 μm (B).
(294 KB PDF)

Figure S3 Floatation Analysis of the Supernatants of Cells Expressing NP Alone or with VP40 (A) Coexpression of NP and VP40 led to the detection of both proteins in the same fractions of the lower sucrose concentrations (fraction number 3).
(B) By contrast, when NP was expressed alone, it was not detected in the supernatant.
(69 KB PDF)

Figure S4 Immunoprecipitation Analysis of the Interaction between VP40 and NP (A) VP40 was expressed alone (VP40) or together with NP (VP40 + NP). Cell lysates and supernatants were immunoprecipitated with an anti-NP antibody and then subjected to Western blotting with an anti-VP40 antibody.
(B) NP was expressed alone (NP) or together with FLAG-tagged VP40 (VP40F). Cell lysates and supernatants were immunoprecipitated with an anti-FLAG antibody and then subjected to Western blotting with an anti-NP antibody.
(685 KB PDF)

Figure S5 Morphologic Changes of Vero E6 Cells upon *Ebolavirus* Infection Vero E6 cells, grown on cover slips, were infected with *Ebolavirus* and observed by SEM. (A) Control Vero E6 cells and (B) virus-infected cells 48 h post-infection. Cell rounding occurs only in virus-infected cells. (B, inset) Enlarged portion of the picture shown by square in (B). The cell surface is covered with numerous horizontally budding virions. Bars, 10 μm.
(1.8 MB PDF)

## Abbreviations

EYFP: enhanced yellow fluorescent protein
GP: glycoprotein
NC: nucleocapsid
NP: nucleoprotein
SEM: scanning electron microscopy
TEM: transmission electron microscopy
VLP: virus-like particle
VP40: viral matrix protein
