# Supplementary figures and images for: Assembly and Budding of Ebolavirus
Source: PLoS Pathog. 2006 Sep 29;2(9):e99. doi: 10.1371/journal.ppat.0020099 (PMC1579243; doi:10.1371/journal.ppat.0020099)

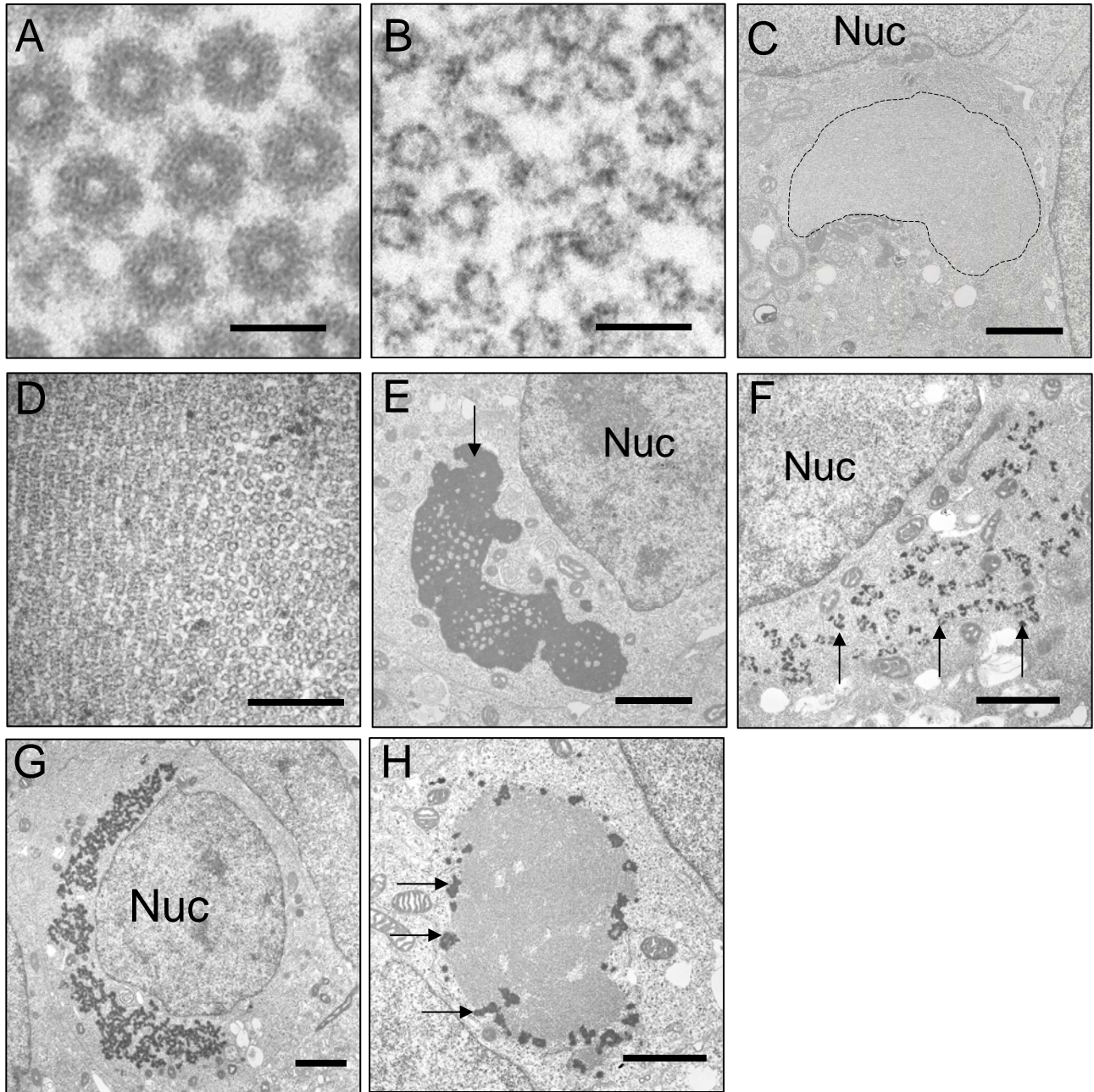

Sup Fig. 1

Supplement: Figure S1 — (A) Coexpression of NP, VP24, and VP35 resulted in the formation of NC-like structures. (B–D) Expression of NP alone produced a mass of helical tubes (C, dotted line) approximately 20–25 nm in diameter near the nucleus (Nuc) (B). Transverse sections of the NP helices are seen on the right (D), while longitudinal sections can be seen on the left (D). (E) Expression of VP35 alone formed large electron-dense aggregates (arrow) near the nucleus (nuc). (F) Expression of VP24 alone resulted in numerous small pleiomorphic structures (arrows) near the nucleus (nuc). (G) Coexpression of VP35 and VP24 produced large structures near nucleus (nuc) that differed from the aggregates formed by expression of either VP24 or VP35 alone. (H) Accumulation of electron-dense aggregates around the mass of NP tubes (arrows) was observed in the cytoplasm of cells coexpressing NP and VP35. Bars, 50 nm (A and B), 2 μm (C, E, G, and H), 500 nm (D), or 1 μm (F). (713 KB PDF) [file ppat.0020099.sg001.pdf]

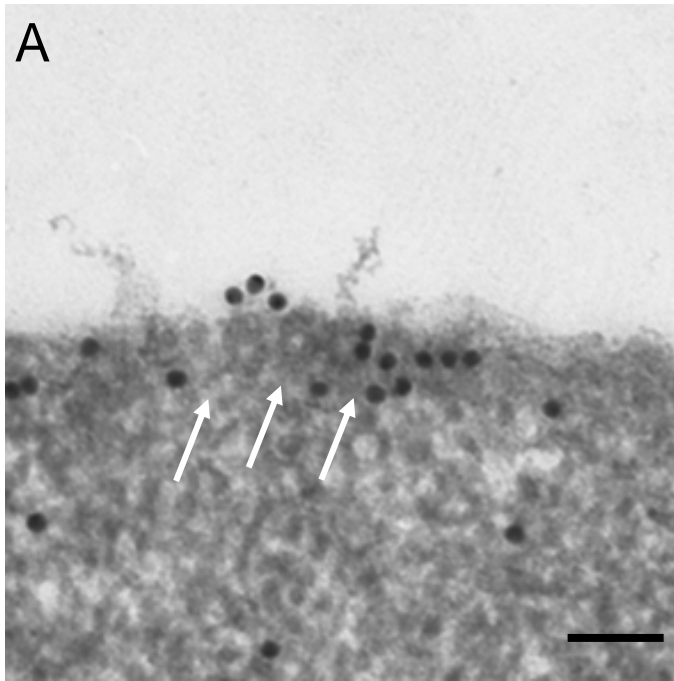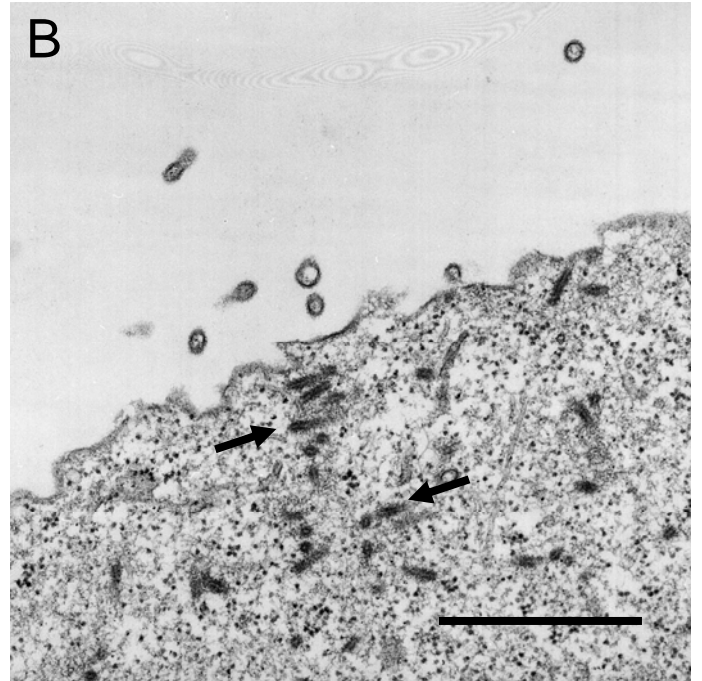

Sup Fig. 2

Supplement: Figure S2 — (A) In cells expressing NP, VP24, VP35, and VP40, VP40 was detected by an anti-VP40 antibody conjugated with 15 nm gold, near to the NC-like structures (white arrows) located beneath the plasma membrane. (B) Upon expression of all viral proteins except VP40 and the minigenome viral RNA, NC-like structures (arrows) remained in the cytoplasm. Bars, 100 nm (A) or 1 μm (B). (294 KB PDF) [file ppat.0020099.sg002.pdf]

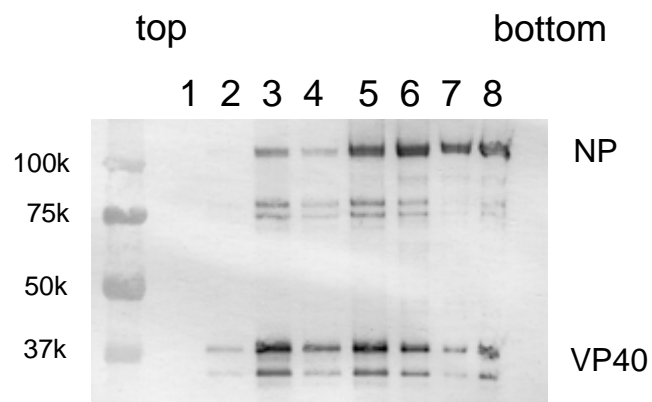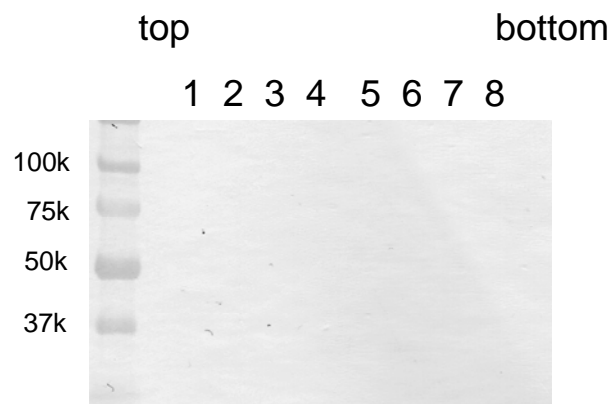

Sup Fig. 3

Supplement: Figure S3 — (A) Coexpression of NP and VP40 led to the detection of both proteins in the same fractions of the lower sucrose concentrations (fraction number 3). (B) By contrast, when NP was expressed alone, it was not detected in the supernatant. (69 KB PDF) [file ppat.0020099.sg003.pdf]

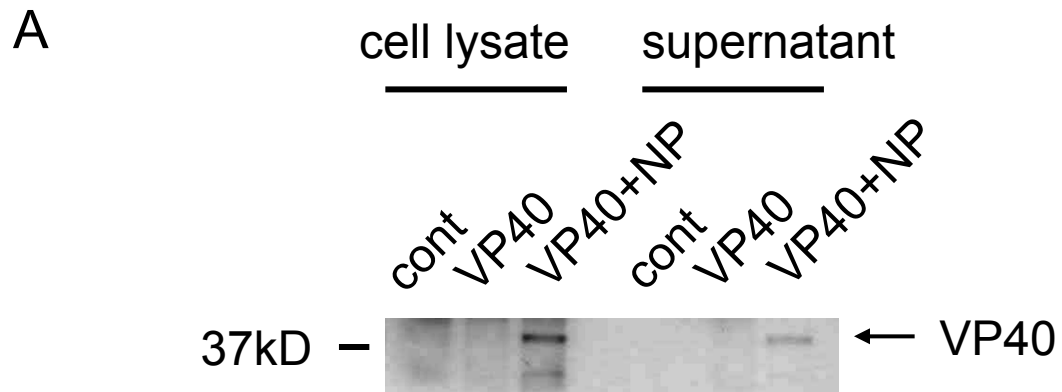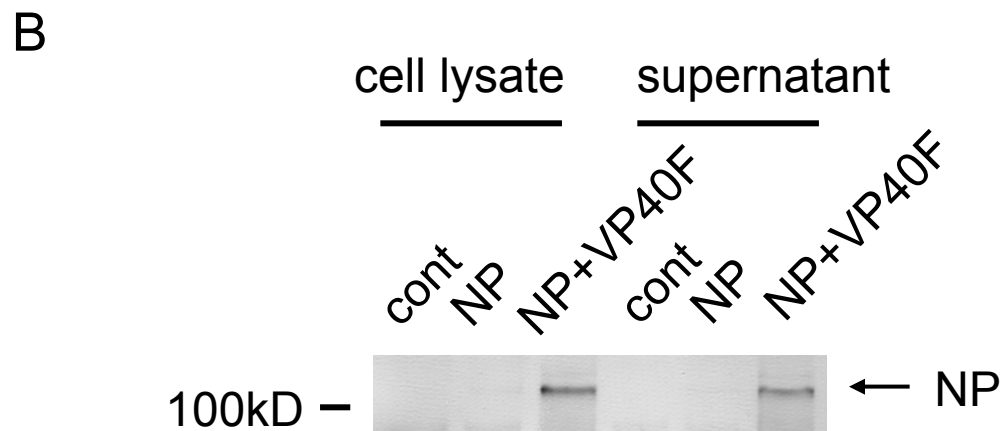

Sup Fig. 4

Supplement: Figure S4 — (A) VP40 was expressed alone (VP40) or together with NP (VP40 + NP). Cell lysates and supernatants were immunoprecipitated with an anti-NP antibody and then subjected to Western blotting with an anti-VP40 antibody. (B) NP was expressed alone (NP) or together with FLAG-tagged VP40 (VP40F). Cell lysates and supernatants were immunoprecipitated with an anti-FLAG antibody and then subjected to Western blotting with an anti-NP antibody. (685 KB PDF) [file ppat.0020099.sg004.pdf]

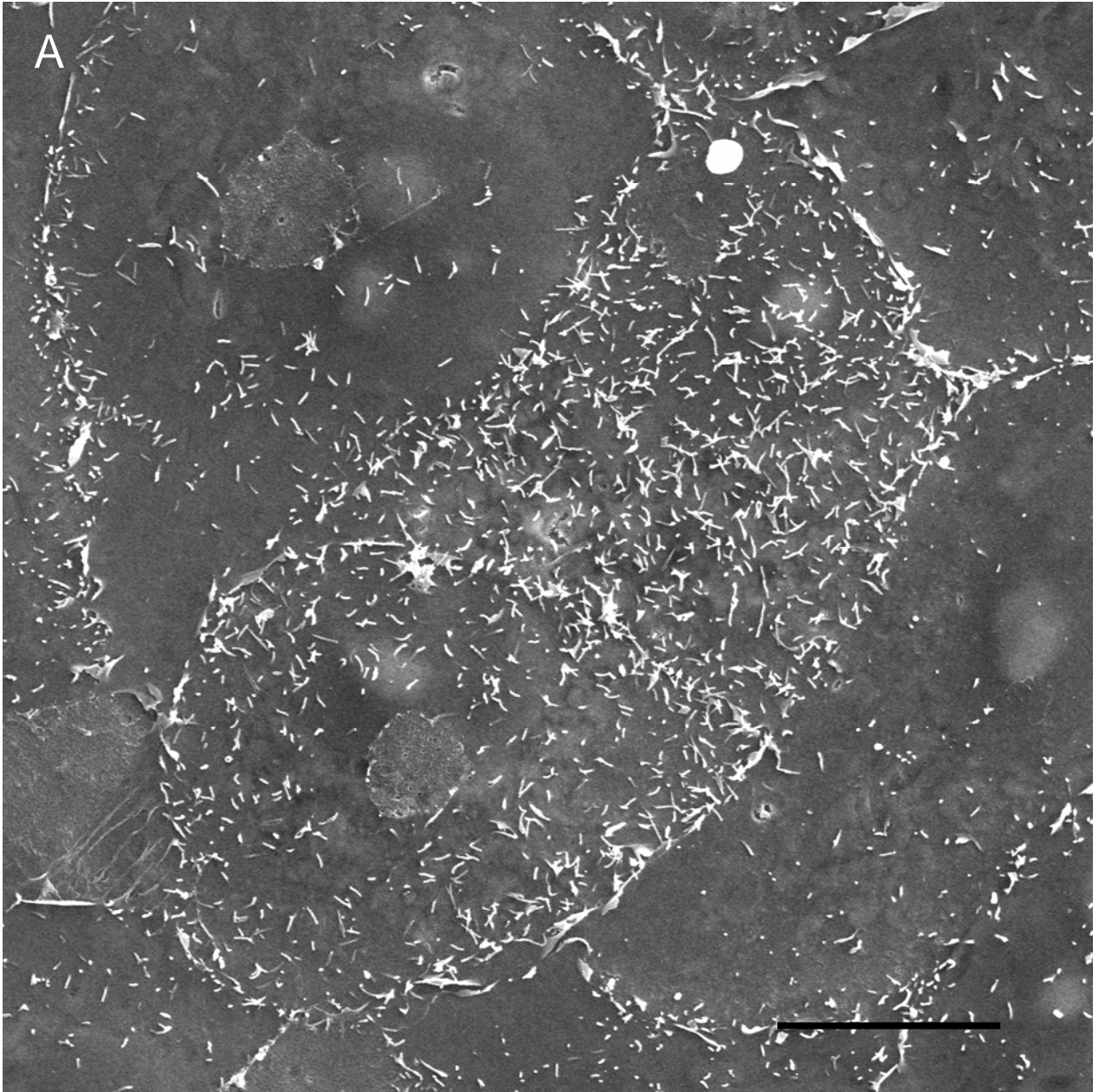

Sup Fig. 5

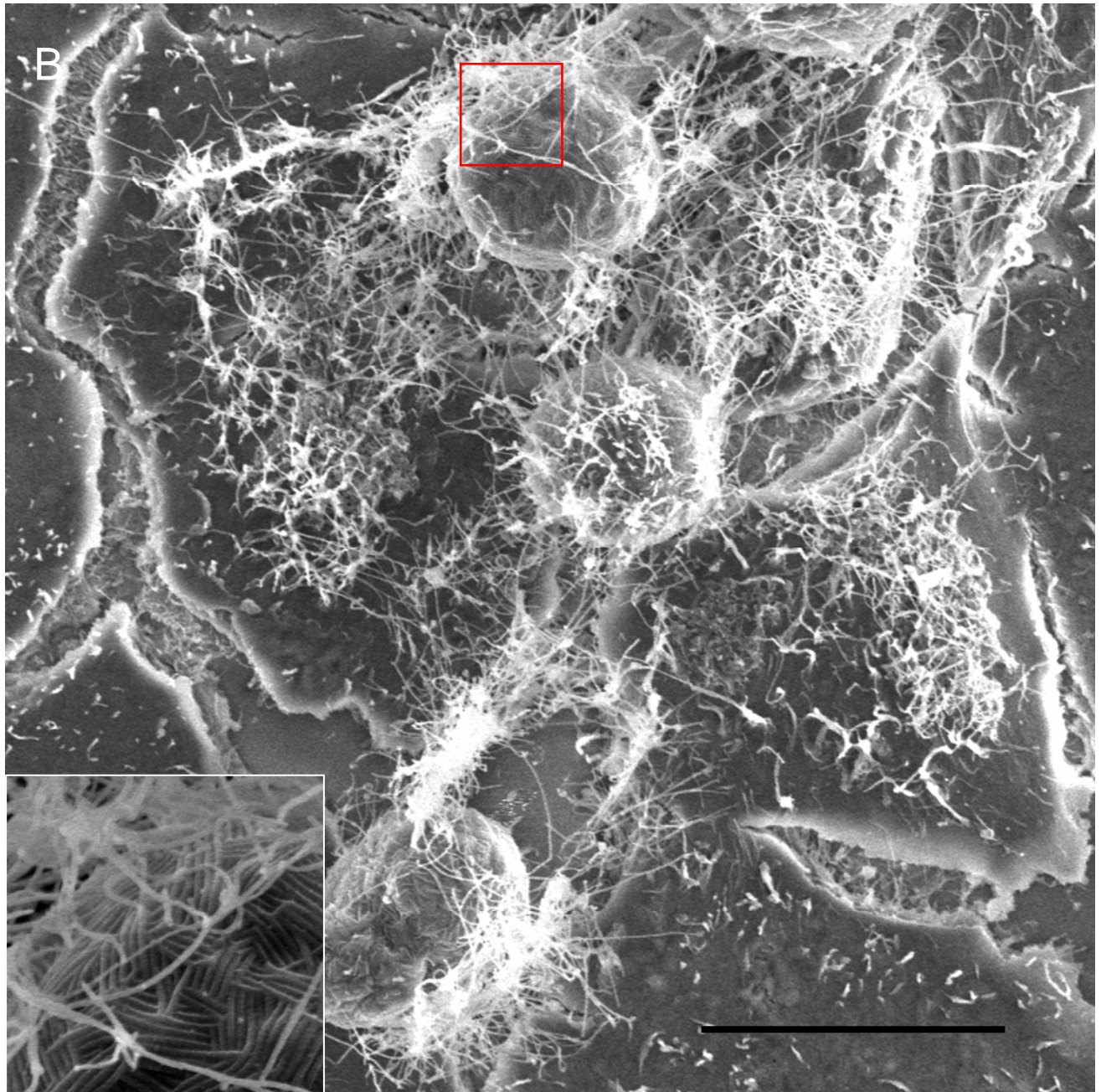

Sup Fig. 5

Supplement: Figure S5 — Vero E6 cells, grown on cover slips, were infected with Ebolavirus and observed by SEM. (A) Control Vero E6 cells and (B) virus-infected cells 48 h post-infection. Cell rounding occurs only in virus-infected cells. (B, inset) Enlarged portion of the picture shown by square in (B). The cell surface is covered with numerous horizontally budding virions. Bars, 10 μm. (1.8 MB PDF) [file ppat.0020099.sg005.pdf]
